# Supplementary material for: Factors affecting revisit intention for medical services at dental clinics
Source: PLoS One. 2021 May 4;16(5):e0250546. doi: 10.1371/journal.pone.0250546 (PMC8096099; doi:10.1371/journal.pone.0250546)
Supplement: S1 Appendix — (DOCX) [file pone.0250546.s001.docx]

S1 Appendix. Questionaire.

| **Communication with the dentist** | Not important at all | | Average | Very important | |
| --- | --- | --- | --- | --- | --- |
| 1. The dentist is willing to listen to me for enough time. | ① | ② | ③ | ④ | ⑤ |
| 2. The dentist explains to me the details of the treatment (diagnosis results, treatment process, and prescription). | ① | ② | ③ | ④ | ⑤ |
| 3. The dentist is polite and friendly when facing patients. | ① | ② | ③ | ④ | ⑤ |
| 4. The dentist creates the atmosphere for me to feel comfortable talking. | ① | ② | ③ | ④ | ⑤ |
| 5. I can ask the dentist, who answers me in an easy way that I could understand for the question. | ① | ② | ③ | ④ | ⑤ |
| 6. The dentist doesn't use easy terms, which I can understand, when explaining. | ① | ② | ③ | ④ | ⑤ |
| 7. The dentist doesn’t make sure I understood the treatment details. | ① | ② | ③ | ④ | ⑤ |
| **Communication with assistant staff (dental hygienists and counselors)** | Not important at all | | Average | Very important | |
| 8. The assistants usually take enough time to listen to me. | ① | ② | ③ | ④ | ⑤ |
| 9. The assistants explain well medical procedures I should take. | ① | ② | ③ | ④ | ⑤ |
| 10. The assistants are polite and friendly when facing patients. | ① | ② | ③ | ④ | ⑤ |
| 11. The assistants create the atmosphere for me to feel comfortable talking. | ① | ② | ③ | ④ | ⑤ |
| **Dentist’s expertise** | Not important at all | | Average | Very important | |
| 13. The dentist seems to know well about sickness. | ① | ② | ③ | ④ | ⑤ |
| 14. The dentist seems to have tested, dosed, and treated me well. | ① | ② | ③ | ④ | ⑤ |
| 15. The dentist understands well what my needs are. | ① | ② | ③ | ④ | ⑤ |
| **Expertise of assistant staff (dental hygienists and counselors)** | Not important at all | | Average | Very important | |
| 16. The counselors seem to be skilled in handling their office work. | ① | ② | ③ | ④ | ⑤ |
| 17. The dental hygienists proficiently perform operations with skilled techniques. | ① | ② | ③ | ④ | ⑤ |
| 18. The assistants (dental hygienists and counselors) understand what my needs are. | ① | ② | ③ | ④ | ⑤ |
| **Reliability of the office or clinic** | Not important at all | | Average | Very important | |
| 19. This dental office or clinic is really reliable and dependable. | ① | ② | ③ | ④ | ⑤ |
| 20. I feel relieved when I receive medical services from the medical staff at this dental office or clinic. | ① | ② | ③ | ④ | ⑤ |
| 21. The dentist considers me like his or her own family when treating me. | ① | ② | ③ | ④ | ⑤ |
| 22. I feel human trust in the dentist. | ① | ② | ③ | ④ | ⑤ |
| **Responsiveness of the office or clinic** | Not important at all | | Average | Very important | |
| 23. The treatment procedure of this dental office or clinic is not convenient. | ① | ② | ③ | ④ | ⑤ |
| 24. I don’t have to wait long to receive the treatment from the dentist at this dental office or clinic. | ① | ② | ③ | ④ | ⑤ |
| 25. This dental office or clinic doesn’t immediately handle it when a patient raises a complaint about services and requests corrections. | ① | ② | ③ | ④ | ⑤ |
| **Physical characteristics of the office or clinic (tangibility)** | Not important at all | | Average | Very important | |
| 26. This dental office or clinic is equipped with the latest medical facilities and equipment. | ① | ② | ③ | ④ | ⑤ |
| 27. This dental office or clinic has the waiting room and lounge, convenient for use by patients or guardians. | ① | ② | ③ | ④ | ⑤ |
| 28. The parking lot of this dental office or clinic is well equipped. | ① | ② | ③ | ④ | ⑤ |
| 29. The facilities and atmosphere at this dental office or clinic are entirely clean. | ① | ② | ③ | ④ | ⑤ |
| 30. The environment of the dental parlor and treatment room at this dental office or clinic is comfortable. | ① | ② | ③ | ④ | ⑤ |
| 31. The interior of the waiting room at this dental office or clinic is generally good. | ① | ② | ③ | ④ | ⑤ |
| **Accessibility to this office or clinic** | Not important at all | | Average | Very important | |
| 32. This dental office or clinic is located in the convenient place. | ① | ② | ③ | ④ | ⑤ |
| 33. This dental office or clinic is located in the more convenient place, compared to other clinics. | ① | ② | ③ | ④ | ⑤ |
| 34. The cost to visit this dental office or clinic is likely to be low. | ① | ② | ③ | ④ | ⑤ |
| 35. Transportation is convenient to come to this dental office or clinic. | ① | ② | ③ | ④ | ⑤ |
| **Overall satisfaction with this office or clinic** | Not important at all | | Average | Very important | |
| 36. I am satisfied with the decision to use this dental office or clinic. | ① | ② | ③ | ④ | ⑤ |
| 37. I am satisfied with the dental medical services provided by this dental office or clinic. | ① | ② | ③ | ④ | ⑤ |
| 38. I think this dental office or clinic is as good as I expected. | ① | ② | ③ | ④ | ⑤ |
| 39. I am willing to recommend this dental office or clinic to others. | ① | ② | ③ | ④ | ⑤ |
| **Service value for this office or clinic** | Not important at all | | Average | Very important | |
| 40. I was provided with as good medical services as the time I invested. | ① | ② | ③ | ④ | ⑤ |
| 41. I received as good medical services as the expense I invested. | ① | ② | ③ | ④ | ⑤ |
| 42. I have achieved what I wanted via the medical services of this dental office or clinic. | ① | ② | ③ | ④ | ⑤ |
| 43. The medical expenses at this dental office or clinic seem to be charged fairly. | ① | ② | ③ | ④ | ⑤ |
| 44. Given the medical expense and treatment outcome, I think it was a good idea to come to this dental office or clinic. | ① | ② | ③ | ④ | ⑤ |
| **Questions about reuse of this office or clinic** | Not important at all | | Average | Very important | |
| 45. I am willing to continuously use this dental office or clinic. | ① | ② | ③ | ④ | ⑤ |
| 46. The next time when I need to see a dentist, I will come to this dental office or clinic. | ① | ② | ③ | ④ | ⑤ |
| 47. I am willing to maintain a continuous relationship with the dentist at this dental office or clinic. | ① | ② | ③ | ④ | ⑤ |
| 48. If there is no special change, I will continue to use this dental office or clinic. | ① | ② | ③ | ④ | ⑤ |
