# Supplementary material for: Factors affecting revisit intention for medical services at dental clinics
Source: PLoS One. 2021 May 4;16(5):e0250546. doi: 10.1371/journal.pone.0250546 (PMC8096099; doi:10.1371/journal.pone.0250546)
Supplement: Attachment — Submitted filename: Response to reviewers1.docx [file pone.0250546.s002.docx]

Response to Reviewer Comments

I wish to thank the reviewers for their constructive feedback. The reviewers point out some remaining elements requiring modifications or clarifications in order to validate my manuscript for publication. My manuscript was thoroughly reviewed and updated according to these pertinent remarks. I would like to illustrate these modifications and address those discussion points in my response below.

**Point 1:** Please ensure that your manuscript meets PLOS ONE's style requirements, including those for file naming.

**Response 1:** Has confirmed.

**Point 2:** Please include additional information regarding the survey or questionnaire used in the study and ensure that you have provided sufficient details that others could replicate the analyses.

**Response 2:**

Questionnaire (S1 Table) added.

**Point 3:** PLOS requires an ORCID iD for the corresponding author in Editorial Manager on papers submitted after December 6th, 2016. Please ensure that you have an ORCID iD and that it is validated in Editorial Manager.

**Response 3:** Has confirmed.

**Point 4:** We note that you have indicated that data from this study are available upon request. PLOS only allows data to be available upon request if there are legal or ethical restrictions on sharing data publicly.

**Response 4:** Has confirmed.

**Point 5:** If there are ethical or legal restrictions on sharing a de-identified data set, please explain them in detail (e.g., data contain potentially identifying or sensitive patient information) and who has imposed them (e.g., an ethics committee). Please also provide contact information for a data access committee, ethics committee, or other institutional body to which data requests may be sent.

**Response 5:**

The study did not include subjects in vulnerable environments and did not collect or record personally identifiable information using information that is disclosed to individuals. We also did not collect or record sensitive information in accordance with Article 23 of the Privacy Act.

**Point 6:** If there are no restrictions, please upload the minimal anonymized data set necessary to replicate your study findings as either Supporting Information files or to a stable, public repository and provide us with the relevant URLs, DOIs, or accession numbers. Please see http://www.bmj.com/content/340/bmj.c181.long for guidelines on how to de-identify and prepare clinical data for publication. For a list of acceptable repositories, please see <http://journals.plos.org/plosone/s/data-availability#loc-recommended-repositories>.

**Response 6:**

The authors confirm that all data underlying the findings are fully available without restriction. All relevant data are within the manuscript and its Supporting Information files.

**Point 7:** Please clarify the sources of funding (financial or material support) for your study. List the grants or organizations that supported your study, including funding received from your institution.

**Response 7:** No financial support was provided to fund this manuscript.

**Point 8:** State what role the funders took in the study. If the funders had no role in your study, please state: “The funders had no role in study design, data collection and analysis, decision to publish, or preparation of the manuscript.”

**Response 8:**

MC and ML conceptualization, operative project leader. SP and ML performed the statistical analysis, interpreted the data, and helped to draft the manuscript. SP participated in the design of the study and helped to draft the manuscript. ML participated in the data collection and revised manuscript. MC designed the study, was principal investigator, participated in the interpretation of the data and revised the manuscript. All authors have read and approved the final manuscript.

**Point 9:** If any authors received a salary from any of your funders, please state which authors and which funders. If you did not receive any funding for this study, please state: “The authors received no specific funding for this work.”

**Response 9:** The authors received no specific funding for this work.

**Point 10:** Please ensure that you refer to Figure 1 in your text as, if accepted, production will need this reference to link the reader to the figure.

**Response 10:** It's corrected.

**Point 11:** Please include captions for your Supporting Information files at the end of your manuscript, and update any in-text citations to match accordingly. Please see our Supporting Information guidelines for more information: http://journals.plos.org/plosone/s/supporting-information.

**Response 11:** It's corrected.

I hope that the considerable changes made to my research paper coupled with our above arguments will convince the reviewers in reconsidering our manuscript for publication in your journal. I thank you in advance for your kind and thorough attention in the review of my work.

Best regards,
