# Supplementary material for: Factors affecting revisit intention for medical services at dental clinics
Source: PLoS One. 2021 May 4;16(5):e0250546. doi: 10.1371/journal.pone.0250546 (PMC8096099; doi:10.1371/journal.pone.0250546)
Supplement: Attachment — Submitted filename: Response to reviewers1.docx [file pone.0250546.s003.docx]

Response to Reviewer Comments

I wish to thank the reviewers for their constructive feedback. The reviewers point out some remaining elements requiring modifications or clarifications in order to validate my manuscript for publication. My manuscript was thoroughly reviewed and updated according to these pertinent remarks. I would like to illustrate these modifications and address those discussion points in my response below.

**Point 1:** Abstract - Please add EXACT results, and provide P values.

**Response 1: P.2**

The factors influencing service value were reliability (β=0.364, p=0.000), expertise (β=0.319, p=0.000), communication by a doctor (β=0.224, p=0.000) and tangibles (β=0.136, p=0.032). In addition, the factors influencing patient satisfaction were in the order of reliability (β=0.258, p=0.000), tangibility (β=0.192, p=0.000), communication by a doctor (β=0.163, p=0.001) and Expertise (β=0.122, p=0.075). On the other hand, service value (β=0.438, p=0.000) had a positive effect on patient satisfaction, and patient satisfaction (β=0.383, p=0.000) was found to influence dental clinics the reuse intention.

**Point 2:** Intro - Please note that each statement of facts must be accompanied by a reference. See, for example, "The importance of communication in medical services has been highlighted before". Revise thoroughly.

**Response 2:**

This part was removed under reviewer advice from a previous revision. Thank you for reviewing my paper so that it can be published.

**Point 3:** Intro - "Therefore, this study analyzes (...)." You have done this already, right? Please switch to past tense. Revise thoroughly throughout your draft.

**Response 3: P.4**

This study analyzes the effect of health communication and service quality on service value, patient satisfaction, and reuse intention, focusing on dental clinics with high patient interaction.

**Point 4:** Intro - Again, , as recommended previously, please provide a null hypothesis. Remember that H0 must be reasonable and deducible from the foregoing thoughts.

**Response 4: P.4**

To this end, in this study, the null hypothesis is that communication by doctors and assistant staff affects the reuse intention medical institutions with service value and patient satisfaction as parameters. In addition, this study aims to analyze by setting the null hypothesis that the medical services quality affects the reuse intention medical institutions by mediating service value and patient satisfaction.

**Point 5:** Meths - "The research model of this study is as follows:" Again, as recommended previously, you should guide the readers. This means that each figure must be accompanied by text, thus explaining the authors' intention.

**Response 5: P.5**

The research model of this study is as follows (Fig 1). This study attempted to analyze the structural impact relationship between health communication and the intention to reuse medical institutions through patient satisfaction and service value. For this, health communication and medical service quality were used as independent variables, and the reuse intention was used as the dependent variable. In addition, patient satisfaction and service value were used as parameters. Communication by doctors and by assistants was selected as sub-items of health communication, which is an independent variable, and expertise, reliability, tangibility, and accessibility were selected and measured as sub-items of medical service quality.

**Point 6:** Meths - "However, there were many incomplete questionnaires." What does this mean? How many is "many"?

**Response 6: P.6**

However, there were 30 incomplete questionnaires

**Point 7:** Meths - Again, please note that this section is not supposed to provide a literature review. This ha been recommended previously, and the authors obviously do not want to follow this aspect. See "In 1985, a study by Parasuraman et al. on the quality of medical services provided five categories of quality. These quality categories include (...)."

**Response 7:**

This part was removed under reviewer advice from a previous revision. Thank you for reviewing my paper so that it can be published.

**Point 8:** Meths - Same with "In 1992, Cronin and Taylor attempted to measure service quality based on (...)." Please revise thoroughly, and do not mix section contents.

**Response 8:**

This part was removed under reviewer advice from a previous revision. Thank you for reviewing my paper so that it can be published.

**Point 9:** Meths - Please provide manufacturers of the software used.

**Response 9: P.8**

The data analysis was conducted using SPSS 25.0 (SPSS Inc., IBM, Chicago, IL, USA) and Amos 18.0 (SPSS Inc., IBM, Chicago, IL, USA) software.

**Point 10:** Results - Please double check legends of both tables and figures, and revise for readability.

**Response 10: P.13-14**

Table 3.

***p<0.001, S.E=Standard error, T=t-value, β=Standardized coefficients, SMC=Squared Multiple Correlation.

Table4.

***p<0.001, X^2^=Chi-square statistic, DF=Degrees of freedom, TLI=Tucker Lewis index, CFI= Comparative fit index, RMSEA=Root mean square error of approximation.

Table5.

*p<0.1, **p<0.05, ***p<0.001, B=Unstandardized coefficients, β=Standardized coefficients, S.E=Standard error, T=t-value

**Point 11:** Results - Instead of indicating ***, please provide exact P values.

**Response 11: P.13**

| **Factor** | **Path** | **Estimate** | **S.E** | **T** | ***p*-value** | **β** | **SMC** |
| --- | --- | --- | --- | --- | --- | --- | --- |
| Health communication | Doctor1 ← Health communication | 1.000 |  |  |  | 0.773 | 0.599 |
|  | Doctor2 ← Health communication | 1.183 | 0.074 | 16.024 | 0.001 | 0.852 | 0.725 |
|  | Doctor3 ← Health communication | 1.009 | 0.067 | 15.145 | 0.001 | 0.814 | 0.660 |
|  | Doctor4 ← Health communication | 1.099 | 0.072 | 15.298 | 0.001 | 0.821 | 0.674 |
|  | Doctor5 ← Health communication | 1.129 | 0.071 | 15.811 | 0.001 | 0.843 | 0.714 |
|  | Doctor6 ← Health communication | 0.872 | 0.081 | 10.783 | 0.001 | 0.372 | 0.610 |
|  | Doctor7 ← Health communication | 0.886 | 0.082 | 10.772 | 0.001 | 0.371 | 0.609 |
|  | Assistant1 ← Health communication | 1.000 |  |  |  | 0.832 | 0.754 |
|  | Assistant2 ← Health communication | 0.980 | 0.054 | 18.112 | 0.001 | 0.850 | 0.743 |
|  | Assistant3 ← Health communication | 0.999 | 0.053 | 18.931 | 0.001 | 0.874 | 0.763 |
|  | Assistant4 ← Health communication | 1.049 | 0.057 | 18.532 | 0.001 | 0.862 | 0.722 |
|  | Assistant5 ← Health communication | 1.044 | 0.056 | 18.743 | 0.001 | 0.868 | 0.692 |
| Expertise | Expertise1 ← Expertise | 1.000 |  |  |  | 0.839 | 0.703 |
|  | Expertise2 ← Expertise | 0.972 | 0.054 | 18.004 | 0.001 | 0.857 | 0.734 |
|  | Expertise3 ← Expertise | 1.138 | 0.055 | 18.952 | 0.001 | 0.889 | 0.791 |
| Reliability | Reliability1 ← Reliability | 1.000 |  |  |  | 0.896 | 0.802 |
|  | Reliability2 ← Reliability | 1.059 | 0.043 | 24.686 | 0.001 | 0.918 | 0.843 |
|  | Reliability3 ← Reliability | 1.024 | 0.051 | 20.198 | 0.001 | 0.838 | 0.702 |
|  | Reliability4 ← Reliability | 1.046 | 0.051 | 20.312 | 0.001 | 0.840 | 0.706 |
| Tangibility | Tangibility1 ← Tangibility | 1.000 |  |  |  | 0.754 | 0.568 |
|  | Tangibility2 ← Tangibility | 1.153 | 0.085 | 13.611 | 0.001 | 0.765 | 0.585 |
|  | Tangibility3 ← Tangibility | 1.227 | 0.077 | 15.998 | 0.001 | 0.882 | 0.778 |
|  | Tangibility4 ← Tangibility | 1.239 | 0.078 | 15.955 | 0.001 | 0.880 | 0.774 |
|  | Tangibility5 ← Tangibility | 1.306 | 0.086 | 15.130 | 0.001 | 0.839 | 0.705 |
|  | Tangibility6 ← Tangibility | 0.979 | 0.110 | 8.924 | 0.001 | 0.271 | 0.520 |
| Accessibility | Accessibility1 ← Accessibility | 1.000 |  |  |  | 0.902 | 0.813 |
|  | Accessibility2 ← Accessibility | 1.083 | 0.053 | 20.472 | 0.001 | 0.934 | 0.872 |
|  | Accessibility3 ← Accessibility | 0.874 | 0.067 | 13.048 | 0.001 | 0.656 | 0.430 |
|  | Accessibility4 ← Accessibility | 0.605 | 0.081 | 7.493 | 0.001 | 0.271 | 0.520 |
| Service value | Service1 ← Service value | 1.000 |  |  |  | 0.941 | 0.885 |
|  | Service2 ← Service value | 1.014 | 0.034 | 30.273 | 0.001 | 0.927 | 0.859 |
|  | Service3 ← Service value | 0.907 | 0.039 | 22.974 | 0.001 | 0.845 | 0.715 |
|  | Service4 ← Service value | 0.801 | 0.045 | 17.679 | 0.001 | 0.751 | 0.564 |
|  | Service5 ← Service value | 0.932 | 0.039 | 23.868 | 0.001 | 0.858 | 0.736 |
| Patient  satisfaction | Satisfaction1 ← Satisfaction | 1.000 |  |  |  | 0.908 | 0.825 |
|  | Satisfaction2 ← Satisfaction | 1.025 | 0.035 | 28.949 | 0.001 | 0.942 | 0.888 |
|  | Satisfaction3 ← Satisfaction | 1.080 | 0.036 | 29.909 | 0.001 | 0.952 | 0.907 |
|  | Satisfaction4 ← Satisfaction | 1.057 | 0.042 | 25.243 | 0.001 | 0.899 | 0.809 |
| Reuse intention | Reuse1 ← Reuse intention | 1.000 |  |  |  | 0.944 | 0.892 |
|  | Reuse2 ← Reuse intention | 1.023 | 0.026 | 39.650 | 0.001 | 0.972 | 0.945 |
|  | Reuse3 ← Reuse intention | 0.968 | 0.028 | 34.935 | 0.001 | 0.947 | 0.897 |
|  | Reuse4 ← Reuse intention | 0.978 | 0.033 | 29.875 | 0.001 | 0.912 | 0.832 |

***p<0.001, S.E=Standard error, T=t-value, β=Standardized coefficients, SMC=Squared Multiple Correlation.

**Point 12:** Disc - "This differs from the findings of previous studies that state (...)." What studies do you refer to here? Again, please note that each such statement must be accompanied by a reference. - Same with "Furthermore, the results also differed from previous study results stating that (...)." Please note that a reviewer's task is not considered to co-author your manuscript.

**Response 12: P.16**

These results are shown in Chang et al. (2013), the doctor's communication attitude affects the overall medical satisfaction of patients, and the medical service quality and the patient's reliability are similar to the results of a study that affects patient satisfaction. Also, Rashid et al. The health communication of doctors in (2014) were similar to the results of a study that showed greater satisfaction to patients than clinical competence [31, 32].

This is, Ehsan et al. (2015) The interaction between the medical staff and the patient affects the overall satisfaction of medical services, and it is different from the research results that the more the communication by doctors and assistant staff with the patients more smoothly, the patient satisfaction is improved [35]. In addition, Fellani Danasra et al. In (2011), most of the patients receiving dental treatment want to talk to the assistant staff about their discomfort, which is different from the research results that have an effect on the patient's reuse intention of dental institutions [36].

**Point 13:** Disc - Same with "This is consistent with the findings of previous studies (...)." Again, this revised and re-submitted draft would not seem convincingly elaborated.

**Response 13: P.16-17**

In Seema (2011), patient satisfaction is similar to the results of a study that observes the treatment process and maintains the continuity of treatment, and that patient satisfaction closely affects the reuse intention a medical institution [37]. Also, Anang et al. In (2019), research results show that the quality of service at a medical institution affects patient satisfaction [38]. Quality of service has a significant effect on the reuse intention and is similar to previous research results that show a significant correlation between satisfaction of outpatients, quality of service, and reuse intention [38-40].

**Point 14:** Concl - Do not repeat methodology or results here. Instead, provide a reasonable extension of your outcome which must stick to the aims of your study.

**Response 14:**

The revision was made according to the reviewer's opinion in the previous revision. Thank you for reviewing my paper so that it can be published.

**Point 15:** Concl - Note that aspects like "limitations" must be given with the Discussion section.

**Response 15:**

The revision was made according to the reviewer's opinion in the previous revision. Thank you for reviewing my paper so that it can be published.

**Point 16:** Refs - Still the references would not seem to follow the Journal's Guidelines for Authors. No doubt, it would seem hard to understand why the authors do not want to follow those guidelines.

**Response 16: P.19**

Modified according to reviewer's advice. Thank you for reviewing my paper so that it can be published.

I hope that the considerable changes made to my research paper coupled with our above arguments will convince the reviewers in reconsidering our manuscript for publication in your journal. I thank you in advance for your kind and thorough attention in the review of my work.

Best regards,
