# Supplementary material for: Factors affecting revisit intention for medical services at dental clinics
Source: PLoS One. 2021 May 4;16(5):e0250546. doi: 10.1371/journal.pone.0250546 (PMC8096099; doi:10.1371/journal.pone.0250546)
Supplement: Attachment — Submitted filename: Reviewer4.docx [file pone.0250546.s004.docx]

Response to Reviewer Comments

I wish to thank the reviewers for their constructive feedback. The reviewers point out some remaining elements requiring modifications or clarifications in order to validate my manuscript for publication. My manuscript was thoroughly reviewed and updated according to these pertinent remarks. I would like to illustrate these modifications and address those discussion points in my response below.

**Point 1:** The manuscript presents extremely relevant data for the organization of health actions; however, some aspects need to be better described. The title of the manuscript leads us to the understanding of the services offered in dental clinics, however, when reading the text there is little focus specifically on this service, since the text is more centered on doctor and medical services. The suggestion is that the text be described about health services in general, and specifically about dental clinics and the dentist. The research was carried out on dental clinics and dentists, however, there is little presence of these terms throughout the text, which allows for a confused reading of the text by over-mentioning the terms doctor and medical services.

**Response 1: P.3**

In general, in order to receive care in Korea, primary and secondary medical institutions must be visited first, and in case of major ailment patients, a medical referral form is issued and care can be received at tertiary medical institutions. However, most dental clinics are composed of primary and secondary medical institutions, and primary medical institutions can receive treatment at the level of tertiary medical institutions. In addition, most of the dental clinics tend to have outpatients and relatively few patients with severe diseases. Therefore, it is necessary to increase the patient's reuse intention through patient-centered communication because the patient can choose a dental clinics to receive treatment [3].

**Point 2:** The abstract needs to present the context of dental clinics and not medical services, it is important to highlight the object of the study. It is necessary to review the verbs and present them in the past. The last sentence of the methods is incomplete. Replace p=0.000 with p<0.001, considering that there is no statistical significance equal to zero. In the statistical packages, when checking the output, it is possible to verify the exact significance.

**Response 2: P.2**

***Abstract***

**Introduction**

Recent changes in the medical paradigm are highlighting the importance of patient-centered communication. However, due to the lack of awareness of dental clinics and of competence in medical personnel, the quality of medical services regarding communication between doctors and patients has not improved. This study analyzes the impact of health communication and medical service quality, service value, and patient satisfaction on revisiting intention for dental clinics.

**Methods**

The study subjects were outpatients who were treated at 10 dental clinics in Seoul. The research data were collected using a questionnaire visited the dental clinics from December 1 to December 30, 2016. A total of 600 questionnaires were distributed to 10 dental clinics, 60 copies each, and 570 valid questionnaires were used for analysis. In this study, the structural influence of factors was determined using structural equation modeling.

**Results**

The factors influencing service value were reliability (β = 0.364, p < 0.001), expertise (β = 0.319, p < 0.001), communication by a doctor (β = 0.224, p < 0.001) and tangibles (β = 0.136, p < 0.05). In addition, the factors influencing patient satisfaction were in the order of reliability (β = 0.258, p < 0.001), tangibility (β = 0.192, p < 0.001), communication by a doctor (β = 0.163, p < 0.001) and Expertise (β = 0.122, p < 0.01). On the other hand, service value (β = 0.438, p < 0.001) had a positive effect on patient satisfaction, and patient satisfaction (β = 0.383, p < 0.001) was found to influence dental clinics the reuse intention.

**Point 3:** Introduction
The first paragraph has no reference. Review throughout the text to prioritize the use of the terms: dentists and dental clinics. It is necessary to review the null hypothesis presented since this hypothesis generally states that there is no relationship between the studied phenomena. The objectives need to be better described, as there is duplication in the presentation.

**Response 3: P.3**

Medical services are changing from a disease-centered model to a patient-centered model. In the existing disease-centered model, all the decision-making concerning patient care was conducted based on the expertise of doctors and other medical personnel, but in the patient-centered model, the patient actively participates in their treatment process and their needs and preferences are reflected in care-related decision making [1, 2].

To this end, the null hypothesis was established that health communication and medical service quality do not affect the reuse intention of dental clinics by mediating service value and patient satisfaction.

**Point 4:** Methods
The text does not make it clear what the inclusion criteria were. Were people under 18 included?
The data analysis section needs to be reviewed carefully. It is necessary to describe in detail the analyzes carried out, as well as the criteria used for each type of analysis.

**Response 4: P.6**

We focused on patients waiting to make a payment or to receive their prescription after receiving treatment as an outpatient over 13 years old in the dental clinic. Since oral care can lead to chronic diseases, regular checkups, and prevention are required from adolescence. The quality of dental services felt by patients during their adolescence can lead to adults, which can be an obstacle to continuous visits to dental institutions, so adolescent patients were included in the study.

**Point 5:** What were the criteria and procedures adopted for the factor analysis? What were the post-tests used to assess the adequacy of the model? What criteria are used?

**Response 5: P.7**

Second, factor analysis was performed to verify the validity of the questions, while the reliability of the measurement questions was validated using Cronbach's alpha (α). In the case of factor analysis, first, an Exploratory Factor Analysis (EFA) of the Varimax mode orthogonal rotation was performed to examine the factor structure of the questions for measuring variables. Next, a Confirmatory Factor Analysis (CFA) was conducted to confirm whether the derived factor structure was consistent with actual empirical data.

**Point 6:** The results related to factor analysis were not presented. What were the communalities, the sample adequacy measures, the variance explained by each factor?

**Response 6: P.10**

An EFA was conducted based on the collected data to examine the factor structure of Forty-eight questions used to measure variables. Factor analysis was carried out by removing the items that hinder the validity and deleting the questions with the lowest commonality. After that, the question with the lowest commonality was deleted and the work of factor analysis was repeated. As a result, the items of expertise of assistant staff, responsiveness of the office or clinic had low commonality and were deleted, and finally, forty-two questions were selected. EFA was again conducted to examine the factor structure of the final selected items. The Karser Meyer Olkin (KMO) = 0.944, Bartlett's test of sphericity test was also significant (χ₂ = 13748.522, P < 0.001), and the data used in the analysis were judged to be suitable for factor analysis. In addition, the Total Variance Explained was 74%.

**Point 7:** In the instrument used, there are more dimensions than those shown in Table 2. The dimensions “Expertise of assistant staff” and “Responsiveness of the office or clinic” are not listed in the table. The number of items in the Communication by assistant dimension is different on the table and on the instrument.

**Response 7: P.10**

An EFA was conducted based on the collected data to examine the factor structure of Forty-eight questions used to measure variables. Factor analysis was carried out by removing the items that hinder the validity and deleting the questions with the lowest commonality. After that, the question with the lowest commonality was deleted and the work of factor analysis was repeated. As a result, the items of expertise of assistant staff, responsiveness of the office or clinic had low commonality and were deleted, and finally, forty-two questions were selected. EFA was again conducted to examine the factor structure of the final selected items. The Karser Meyer Olkin (KMO) = 0.944, Bartlett's test of sphericity test was also significant (χ₂ = 13748.522, P < 0.001), and the data used in the analysis were judged to be suitable for factor analysis. In addition, the Total Variance Explained was 74%.

**Point 8:** The instrument contains 48 items; however, it was presented that the analysis was performed with only 38 items, it is necessary to present the reasons that led to the exclusion of 10 items.

**Response 8: P.11**

In the CFA, two items on communication by doctors, one on tangibility, and one on accessibility did not exceed the 0.5 standard factor loading criterion. That is, out of the Forty-eight questions used for data collection, six items with poor commonality were removed through EFA, and four items with poor validity were removed through CFA. Therefore, 38 items—excluding ten items—were used for the analysis. Table 3 shows the results of the CFA for the model used in this study.

**Point 9:** In table 5, it is necessary to review the presentation of the p-values (p=0.000). If the p-value was presented, it is not necessary to use symbols to describe the statistical significance.

**Response 9: P.14**

Table 5. Research model path coefficients.

| **Factor** | **Path** | **B** | **β** | **S.E.** | **T** | ***p*-value** |
| --- | --- | --- | --- | --- | --- | --- |
| Service value | Service ← Communication by doctor | 0.215 | 0.224 | 0.060 | 3.600^**^ | 0.001 |
|  | Service ← Communication by assistant | -0.038 | -0.037 | 0.066 | -0.580 | 0.562 |
|  | Service ← Expertise | 0.321 | 0.319 | 0.086 | 3.748^***^ | 0.001 |
|  | Service ← Reliability | 0.365 | 0.364 | 0.089 | 4.113^***^ | 0.001 |
|  | Service ← Tangibility | 0.175 | 0.136 | 0.081 | 2.145^**^ | 0.032 |
|  | Service ← Accessibility | 0.014 | 0.014 | 0.052 | 0.259 | 0.795 |
| Patient satisfaction | Satisfaction ← Communication by doctor | 0.140 | 0.163 | 0.044 | 3.211^**^ | 0.001 |
|  | Satisfaction ← Communication by assistant | 0.046 | 0.050 | 0.046 | 0.997 | 0.319 |
|  | Satisfaction ← Expertise | 0.110 | 0.122 | 0.062 | 1.778^*^ | 0.075 |
|  | Satisfaction ← Reliability | 0.231 | 0.258 | 0.064 | 3.585^***^ | 0.001 |
|  | Satisfaction ← Tangibility | 0.220 | 0.192 | 0.059 | 3.729^***^ | 0.001 |
|  | Satisfaction ← Accessibility | -0.005 | -0.005 | 0.037 | -0.130 | 0.897 |
|  | Satisfaction ← Service value | 0.397 | 0.444 | 0.050 | 7.941^***^ | 0.001 |
| Reuse intention | Reuse ← Patient satisfaction | 0.491 | 0.383 | 0.087 | 5.616^***^ | 0.001 |
|  | Reuse ← Service value | 0.501 | 0.438 | 0.078 | 6.414^***^ | 0.001 |

**p<0.1, **p<0.05, ***p<0.001, B=Unstandardized coefficients, β=Standardized coefficients, S.E=Standard error, T=t-value*

**Point 10:** In figure 2, the expertise dimension has repeated values, it is necessary to correct it. As this is the figure that presents the final model, I suggest that only the relations that were significant for the composition of the final model be presented.

**Response 10: P.15**

Fig 2. Final path model.

**Point 11:** In view of the notes made, it is suggested that all dimensions of the final model be addressed in the discussion. It is necessary to review the use of the terms doctor and medical services.

**Response 11: P.15-16**

As a result of the study, health communication and quality of medical service influenced the reuse intention of dental clinics by mediating patient satisfaction and service value, thus rejecting the null hypothesis and adopting the alternative hypothesis. The detailed study results are as follows.

**Point 12:** It is highly recommended that the title, objectives and conclusion are related and that the conclusion responds directly to the proposed objectives.

**Response 12: P.18**

This study aimed to analyze the influencing factors of the reuse of medical services, employing data from patients visiting dental clinics located in Seoul. The results showed that reliability and communication by doctors affected service value and patient satisfaction, which had an effect on reuse intention. It is considered that the following measures are necessary to increase the satisfaction of patients who have visiting dental clinics and to increase the reuse intention. Dental clinics should provide appropriate medical services to outpatients, which is based on smooth communication between doctors and patients. Additionally, doctors having an attitude of respect toward the patient may affect patient satisfaction. Doctors providing medical treatment information to patients with a friendly and respectful attitude rather than an authoritarian attitude may be an effective strategy for dental medical institutions to attract long-term customers.

I hope that the considerable changes made to my research paper coupled with our above arguments will convince the reviewers in reconsidering our manuscript for publication in your journal. I thank you in advance for your kind and thorough attention in the review of my work.

Best regards,
