# Supplementary material for: Factors affecting revisit intention for medical services at dental clinics
Source: PLoS One. 2021 May 4;16(5):e0250546. doi: 10.1371/journal.pone.0250546 (PMC8096099; doi:10.1371/journal.pone.0250546)
Supplement: Attachment — Submitted filename: Response to reviewers1.docx [file pone.0250546.s005.docx]

Response to Reviewer Comments

I wish to thank the reviewers for their constructive feedback. The reviewers point out some remaining elements requiring modifications or clarifications in order to validate my manuscript for publication. My manuscript was thoroughly reviewed and updated according to these pertinent remarks. I would like to illustrate these modifications and address those discussion points in my response below.

**Point 1:** Still, some minor typos are evident with the Reference section, and the latter would seem lacking uniformity. These aspects will be revised with the proofs, so please pay special attention to the proof reading. This revised and re-submitted manuscript would seem ready to proceed.

**Response 1:**

Modified it according to the reviewer's advice. Thank you for reviewing my paper so that it can be published.

I hope that the considerable changes made to my research paper coupled with our above arguments will convince the reviewers in reconsidering our manuscript for publication in your journal. I thank you in advance for your kind and thorough attention in the review of my work.

Best regards,
